# Targeting FcγRIIB by antagonistic antibody BI-1206 improves the efficacy of rituximab-based therapies in aggressive mantle cell lymphoma

**DOI:** 10.1186/s13045-022-01257-9

**Published:** 2022-04-11

**Authors:** Vivian Changying Jiang, Yang Liu, Alexa Jordan, Angela Leeming, Joseph McIntosh, Shengjian Huang, Rongjia Zhang, Qingsong Cai, Zhihong Chen, Yijing Li, Yuxuan Che, Lei Nie, Ingrid Karlsson, Linda Mårtensson, Mathilda Kovacek, Ingrid Teige, Björn Frendéus, Michael Wang

**Affiliations:** 1 Department of Lymphoma and Myeloma, The University of Texas MD Anderson Cancer Center, 1515 Holcombe Blvd., Houston, TX USA; 2 BioInvent International AB, Lund, Sweden; 3 Department of Stem Cell Transplantation and Cellular Therapy, The University of Texas MD Anderson Cancer Center, Houston, TX USA

**Keywords:** FcγRIIB, BI-1206, CAR T, Therapeutic resistance, Mantle cell lymphoma, Combination

## Abstract

**Abstract:**

Inevitable relapses remain as the major therapeutic challenge in patients with mantle cell lymphoma (MCL) despite FDA approval of multiple targeted therapies and immunotherapies. Fc gamma receptors (FcγRs) play important roles in regulating antibody-mediated immunity. FcγRIIB, the unique immune-checkpoint inhibitory member of the FcγR family, has been implicated in immune cell desensitization and tumor cell resistance to the anti-CD20 antibody rituximab and other antibody-mediated immunotherapies; however, little is known about its expression and its immune-modulatory function in patients with aggressive MCL, especially those with multi-resistance. In this study, we found that FcγRIIB was ubiquitously expressed in both MCL cell lines and primary patient samples. FcγRIIB expression is significantly higher in CAR T-relapsed patient samples (*p* < 0.0001) compared to ibrutinib/rituximab-naïve, sensitive or resistant samples. Rituximab-induced CD20 internalization in JeKo-1 cells was completely blocked by concurrent treatment with BI-1206, a recombinant human monoclonal antibody targeting FcγRIIB. Combinational therapies with rituximab-ibrutinib, rituximab-venetoclax and rituximab-CHOP also induced CD20 internalization which was again effectively blocked by BI-1206. BI-1206 significantly enhanced the in vivo anti-MCL efficacy of rituximab-ibrutinib (*p* = 0.05) and rituximab-venetoclax (*p* = 0.02), but not the rituximab-CHOP combination in JeKo-1 cell line-derived xenograft models. In patient-derived xenograft (PDX) models, BI-1206, as a single agent, showed high potency (*p* < 0.0001, compared to vehicle control) in one aggressive PDX model that is resistant to both ibrutinib and venetoclax but sensitive to the combination of rituximab and lenalidomide (the preclinical mimetic of *R*^2^ therapy). BI-1206 sensitized the efficacy of rituximab monotherapy in a PDX model with triple resistance to rituximab, ibrutinib and CAR T-therapies (*p* = 0.030). Moreover, BI-1206 significantly enhanced the efficacy of the rituximab-venetoclax combination (*p* < 0.05), which led to long-term tumor remission in 25% of mice. Altogether, these data support that targeting this new immune-checkpoint blockade enhances the therapeutic activity of rituximab-based regimens in aggressive MCL models with multi-resistance.

**Graphical Abstract:**

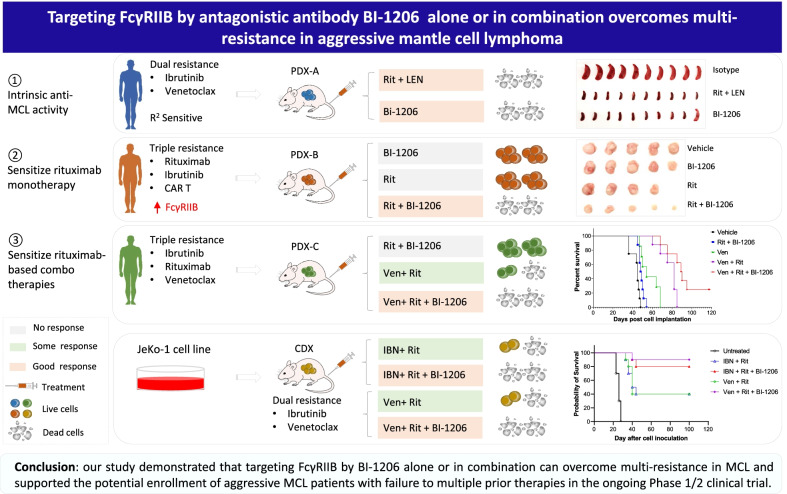

**Supplementary Information:**

The online version contains supplementary material available at 10.1186/s13045-022-01257-9.

## To the Editor,

FcγRIIB is the only inhibitory member of the FcγR immunomodulator family by negatively regulating antibody-based immune cytotoxicity [1]. FcγRIIB promotes rituximab-CD20 internalization and confers therapeutic resistance to rituximab in B cell lymphoma [2, 3]. Therefore, targeting FcγRIIB with specific antibodies has the potential to promote the efficacy of other antibody-based immunotherapies. Anti-FcγRIIB antibody BI-1206, a recombinant human monoclonal antibody, was developed to prevent FcγRIIB-mediated CD20-rituximab internalization [4].

**Fig. 1:**
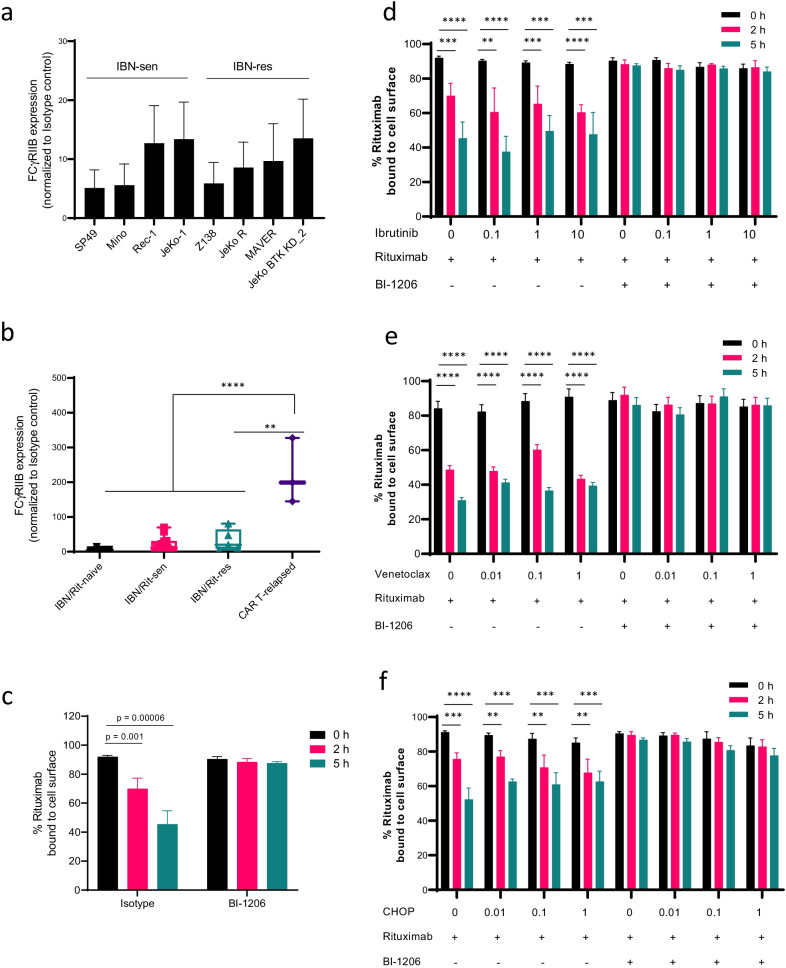
FcγRIIB is ubiquitously expressed in MCL cells and targeting FcγRIIB by BI-1206 effectively blocks CD20 internalization upon rituximab-based treatment in MCL cells. **a**, **b** Flow cytometry analysis was performed to detect FcγRIIB expression on MCL cell lines (**a**) and primary patient MCL cells (**b**) including ibrutinib/rituximab-naïve (*n* = 4), ibrutinib/rituximab-sensitive (*n* = 13), ibrutinib/rituximab-resistant (*n* = 5) and CAR T-relapsed (*n* = 3) MCL samples. **c** Percentage of rituximab bound CD20 on JeKo-1 cells upon treatment with rituximab (5 μg/ml) with or without BI-1206 (5 μg/ml) for 0, 2 and 5 h. **d–f** Percentage of rituximab bound CD20 on JeKo-1 cells after 48 h pre-treatment in vitro with increasing concentrations of ibrutinib (**d**), venetoclax (**e**) or CHOP (**f**) followed by treatment with rituximab with or without BI-1206 for 0, 2 and 5 h (Mean ± SD; n = 4). *P < 0.05; **P < 0.01; ***P < 0.001; ****P < 0.0001

We detected ubiquitous FcγRIIB expression in all 8 MCL cell lines (100%) and in 24/25 (96%) primary MCL patient samples (Fig. 1a). Elevated FcγRIIB expression is associated with CAR T-relapse in MCL (*n* = 3, *p* < 0.0001) but not with ibrutinib/rituximab resistance (Fig. 1b and Additional file 1: Figure S1). Rituximab-induced CD20 internalization [3, 5] was effectively blocked by BI-1206 in JeKo-1 cells (Fig. 1c). In line with this, rituximab-based combinations, including rituximab-ibrutinib, rituximab-venetoclax or rituximab-CHOP, also induced CD20 internalization. Importantly, concurrent BI-1206 treatment targeting FcγRIIB effectively blocked FcγRIIB-mediated CD20 internalization in vitro that was induced by these rituximab-based combinations (*p* < 0.01) at 2 and 5 h post-treatment (Fig. 1d–f).

**Fig. 2:**
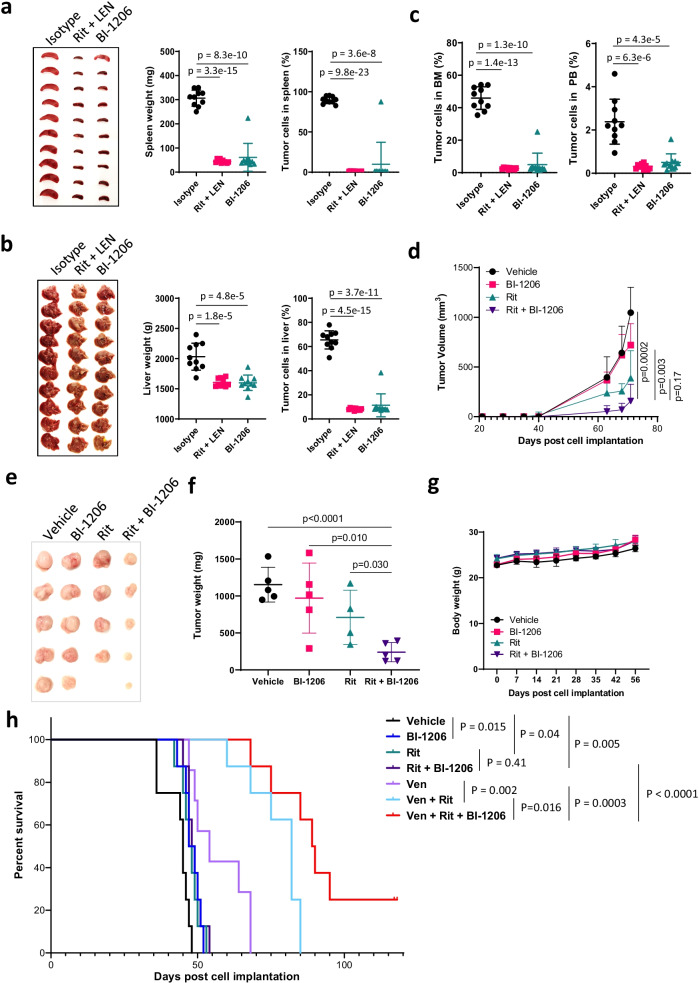
BI-1206 enhanced the in vivo efficacy of rituximab-based therapy in various MCL PDX models with multi-resistance in addition to intrinsic anti-MCL activity. **a**–**c** An ibrutinib-venetoclax dual resistant MCL PDX model (PDX-A) was derived from a patient with MCL with subsequent relapse from ibrutinib and venetoclax therapy. The PDX cells were inoculated intravenously into NOD.Cg-*Prkdc*^*scid*^* Il2rg*^*tm1Wjl*^/SzJ (NSG) mice. At 2 weeks post-cell inoculation, the mice (*n* = 10 per group) were randomly grouped and treated with isotype control (10 mg/kg, twice a week), BI-1206 (10 mg/kg, twice a week) or Rituximab (Rit, 10 mg/kg, twice a week) plus Lenalidomide (LEN, 2 mg/kg, daily). At the end of the experiment, the mice were euthanized, and spleen, liver, bone marrow and peripheral blood were collected. **a**, **b** The mouse spleen (**a**) and liver (**b**) were imaged (left panels) and weighed (middle panels). The cells in the spleen and liver were isolated and subject to flow analysis by dual staining with CD5 and CD20 antibodies. The percentage of CD5^+^CD20^+^ cells representing MCL tumor cells were plotted (right panels). **c** The cells in the bone marrow (left panel) and peripheral blood (right panel) were isolated and subject to flow analysis by dual staining with CD5 and CD20 antibodies. The percentage of CD5^+^CD20^+^ cells representing MCL tumor cells were plotted. **d–g** A rituximab-ibrutinib-CAR T-triple resistant MCL PDX model (PDX-B) was derived from a patient with MCL with subsequent relapse from rituximab, ibrutinib and CAR T-therapy. The PDX cells were inoculated subcutaneously into NSG mice. At 2 weeks post-cell inoculation, the mice (*n* = 5 per group) were randomly grouped and treated with isotype control (10 mg/kg, twice a week), BI-1206 (10 mg/kg, twice a week), Rituximab (Rit, 10 mg/kg, twice a week) or Rit + BI-1206 combo. One mouse in the rituximab-treated group died due to treatment-irrelevant technical event. Tumor size (**d**) and mouse body weight (**g**) were measured every week. At the end of the experiment, the mice were euthanized, and subcutaneous tumors were collected, imaged (**e**) and weighed (**f**). **h** An ibrutinib-rituximab-venetoclax triple-resistant MCL PDX (PDX-C) model was derived from a patient with MCL with relapse from ibrutinib plus rituximab therapy and venetoclax therapy. The PDX cells were inoculated subcutaneously into NSG mice. At 2 weeks post-cell inoculation, the mice (*n* = 8 per group) were randomly grouped and treated with vehicle control, BI-1206 (10 mg/kg, twice a week), Rituximab (Rit, 10 mg/kg, twice a week), Rituximab + BI-1206 (Rit + BI-1206), venetoclax (Ven, 50 mg/kg, daily), venetoclax + Rituximab (Ven + Rit) or venetoclax + Rituximab + BI-1206 (Ven + Rit + BI-1206). Mouse survival was monitored and plotted

We then assessed the anti-tumor cytotoxicity of BI-1206 in vivo in CDX or PDX models. In the disseminated PDX model (PDX-A) with resistance to ibrutinib and venetoclax, the tumor cells dissemiated to the spleen, liver, bone marrow (BM), peripheral blood (PB) and 0–2 lymph nodes but not to the lungs or kidneys (Additional file 2: Figure S2a, S2e-g). Strikingly, BI-1206 and the combination of rituximab and lenalidomide (Revlimid) (the preclinical mimetic of *R*^2^ therapy) were both highly potent in blocking tumor growth (*p* < 0.0001) in this *R*^2^-sensitive PDX-A model (Fig. 2a–c and Additional file 2: Figure S2c-d). Next, we investigated whether BI-1206 can sensitize MCL tumors to rituximab by leveraging a rituximab-resistant PDX model (PDX-B) which was derived from a patient post-subsequent failure to rituximab, ibrutinib and CAR T-therapy. BI-1206 alone did not show significant anti-MCL efficacy in PDX-B, but when combined with rituximab, it significantly inhibited tumor growth compared to either single agent (*p* < 0.05) (Fig. 2d–f). This was consistent with the pilot data from the clinical trial NCT03571568, which showed BI-1206 enhanced rituximab efficacy in two-thirds of rituximab-refractory/relapsed (R/R) patients [6].

In order to further improve the BI-1206-rituximab efficacy, we evaluated the potential of using them in combination with targeted therapies beginning in JeKo-1 CDX models in vivo*.* The triple combination (*n* = 10 per group) with ibrutinib or venetoclax, but not with CHOP, showed significantly better efficacy and prolonged mouse survival compared to the dual combinations without BI-1206 (Additional file 3: Figure S3). To address this further, we assessed the in vivo efficacy of the BI-1206-rituximab-venetoclax combination in a PDX model (PDX-C) with triple resistance to rituximab, ibrutinib and venetoclax. The BI-1206-rituximab combination is no longer effective in PDX-C, but when combined with venetoclax, it showed superior anti-tumor activity and prolonged mouse survival compared to any single or dual combination (*p* < 0.05) (Fig. 2h). Together, these data demonstrated that the BI-1206-rituximab-venetoclax triple combination achieved the best efficacy across all treatment arms and thus provides insights into future therapeutic implementation of combinational therapies as precision medicine. In addition, no unanticipated adverse events were observed in any of the mice treated with BI-1206 alone or in combination (Fig. 2g and Additional file 2: Figure S2b).

Novel therapies to overcome therapeutic resistance in R/R patients with MCL are in urgent need, particularly for patients relapsed from CAR-T therapy. In this study, we observed ubiquitous expression of FcγRIIB in MCL cells and elevated levels in CAR-T-relapsed patients with MCL. By leveraging various multi-resistant PDX models, we demonstrated that BI-1206 monotherapy or its rational combination with various clinically approved drugs such as rituximab, ibrutinib or venetoclax could effectively improve their efficacy. Due to inter-tumor heterogeneity and diverse multi-resistance, each MCL tumor may rely on different set of determinants for their survival and growth; therefore, it is critical to match tailored treatments for these aggressive MCL tumors. Altogether, our data provide evidence for further preclinical investigation and potential clinical use of BI1206-based therapy for aggressive MCL tumors with multi-resistance. With the exciting proof of concept pilot clinical data for the BI-1206-rituximab combination [7], and our preclinical data in this study, a cohort of patients with multiple therapeutic failures is being considered to be enrolled in the clinical trial.

## Supplementary Information

**Additional file 1**. **Supplementary Figure S1.** FcγRIIB and CD20 expression in MCL cells. Flow cytometry analysis was performed to detect FcγRIIB expression in primary patient MCL cells including ibrutinib-naïve (n = 4), ibrutinib-sensitive (n = 13), ibrutinib-resistant (n = 5) and CAR T-relapsed (n = 3) MCL samples.

**Additional file 2**. **Supplementary Figure S2.** Efficacy assessment of BI-1206 monotherapy in an ibrutinib-venetoclax dual resistant MCL PDX model in vivo. (a) Schematic strategy of the experiment to assess in vivo efficacy in the PDX model. PDX-A cells were inoculated intravenously into NSG mice. At two weeks post cell inoculation, the mice (n = 10 per group) were randomly grouped and treated with isotype control (10 mg/kg, twice a week), BI-1206 (10 mg/kg, twice a week) or Rituximab (Rit, 10 mg/kg, twice a week) plus Lenalidomide (LEN, 2 mg/kg, daily). Peripheral blood was collected every two weeks. At the end of the experiment, the mice were euthanized, and peripheral blood was collected. (b) The mouse body weight was monitored every two weeks. (c-d) Peripheral blood was collected every two weeks and at the end of experiment, and subject to flow analysis by dual staining with CD5 and CD20 antibodies. The percentage of CD5+CD20+ cells representing MCL tumor cells were plotted (c). Human B2M levels in mouse serum were detected via B2M ELISA assay. (e-f) Lung (e) and kidney (f) were weighed. (g) Counts of lymph nodes were plotted.

**Additional file 3**. **Supplementary Figure S3.** BI-1206 enhanced in vivo anti-MCL efficacy of ibrutinib + rituximab or venetoclax + rituximab combinations. (a-b) JeKo-1 tumor volume in untreated animals (circles), after treatment with rituximab and ibrutinib (IBN + Rit, squares), or with rituximab and ibrutinib in combination with BI-1206 (IBN + Rit + BI-1206, triangles) at day 14 after treatment initiation. Ibrutinib (12.5 mg/kg) was given daily for 14 days. Rituximab (10 mg/kg) and BI-1206 (10 mg/kg) were given twice a week for 14 days. Treatment was initiated at day 14 post inoculation. Tumor volumes were measured at day 14 post treatment and mouse survival was monitored and plotted. Student t test and Log-rank test were used to generate p values comparing the dual and triple combinations. n = 10 mice per group per treatment arm. (c-d) JeKo-1 tumor volume in untreated mice (circles), after treatment with rituximab and venetoclax (Ven + Rit, squares) or with rituximab and venetoclax in combination with BI-1206 (Ven + Rit + BI-1206, triangles) at day 14 after treatment initiation. Venetoclax (100 mg/kg) was given daily for 14 days. Rituximab (10 mg/kg) and BI-1206 (10 mg/kg) were given twice a week for 14 days. Treatment was initiated at day 14 post inoculation. Tumor volumes were measured at day 14 post treatment and mouse survival was monitored and plotted. Student t test and Log-rank test were used to generate p values comparing the dual and triple combos. n = 10 mice per group per treatment arm. (e-f) JeKo-1 tumor volume in untreated animals (circles), after treatment with rituximab and CHOP (CHOP + Rit, squares) or with rituximab and CHOP in combination with BI-1206 (CHOP + Rit + BI-1206, triangles) at day 8 after treatment initiation. For CHOP treatment, cyclophosphamide (C; 20 mg/kg), hydroxy doxorubicin (H; 1.65 mg/kg), oncovin (O; 0.25 mg/kg) was given daily for 14 days and prednisone (P; 0.1 mg/kg) was given 5 days per week for 14 days. Rituximab (10 mg/kg) and BI-1206 (10 mg/kg) were given twice a week for 14 days. Treatment was initiated at day 21 post inoculation. Student t test and log-rank test were used to generate p values comparing the dual and triple combos. n = 10 mice per group per treatment arm.

## Data Availability

All data generated or analyzed during this study are included in this published article and its supplementary information files.

## Abbreviations

BM: Bone marrow
CAR: Chimeric antigen receptor
CDX: Cell line-derived xenograft
FcγRIIB: Fc gamma receptor II B
FcγRs: Fc gamma receptors
IBN: Ibrutinib
i.v.: Intravenously
LEN: Lenalidomide
MCL: Mantle cell lymphoma
NSG: NOD.Cg-*Prkdc*^*scid*^* Il2rg*^*tm1Wjl*^/SzJ
PB: Peripheral blood
PDX: Patient-derived xenograft
Rit: Rituximab
R/R: Refractory or relapsed
Ven: Venetoclax
