# Supplementary figures and images for: Targeting FcγRIIB by antagonistic antibody BI-1206 improves the efficacy of rituximab-based therapies in aggressive mantle cell lymphoma
Source: J Hematol Oncol. 2022 Apr 11;15:42. doi: 10.1186/s13045-022-01257-9 (PMC8996600; doi:10.1186/s13045-022-01257-9)

## Slide 1
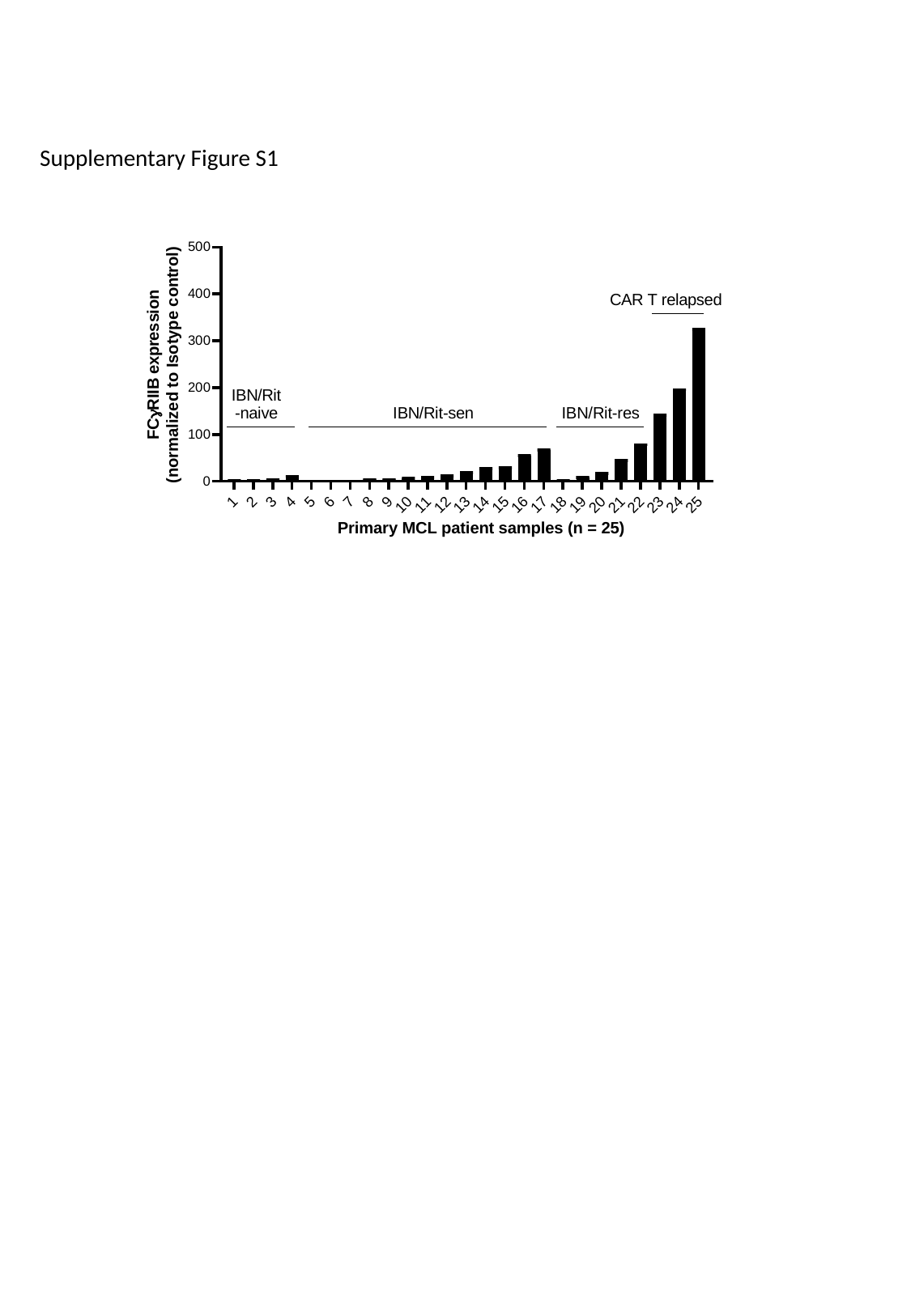

Supplementary Figure S1

Supplement: Supplementary file 1 — Additional file 1. Supplementary Figure S1. FcγRIIB and CD20 expression in MCL cells. Flow cytometry analysis was performed to detect FcγRIIB expression in primary patient MCL cells including ibrutinib-naïve (n = 4), ibrutinib-sensitive (n = 13), ibrutinib-resistant (n = 5) and CAR T-relapsed (n = 3) MCL samples. [file 13045_2022_1257_MOESM1_ESM.pptx]

## Slide 1
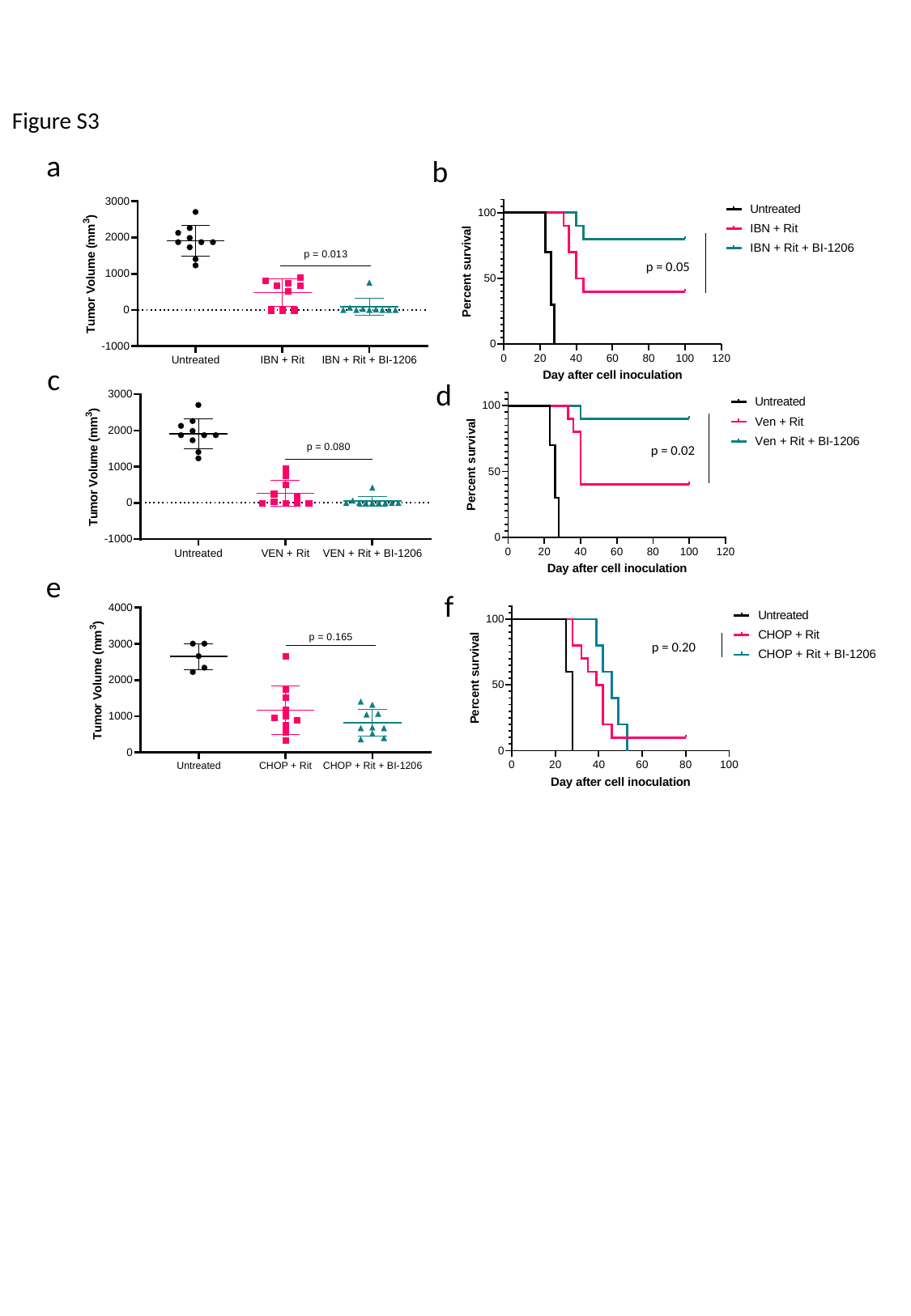

Figure S3
a
b
p = 0.05
p = 0.02
p = 0.20
c
d
e
f

Supplement: Supplementary file 3 — Additional file 3. Supplementary Figure S3. BI-1206 enhanced in vivo anti-MCL efficacy of ibrutinib + rituximab or venetoclax + rituximab combinations. (a-b) JeKo-1 tumor volume in untreated animals (circles), after treatment with rituximab and ibrutinib (IBN + Rit, squares), or with rituximab and ibrutinib in combination with BI-1206 (IBN + Rit + BI-1206, triangles) at day 14 after treatment initiation. Ibrutinib (12.5 mg/kg) was given daily for 14 days. Rituximab (10 mg/kg) and BI-1206 (10 mg/kg) were given twice a week for 14 days. Treatment was initiated at day 14 post inoculation. Tumor volumes were measured at day 14 post treatment and mouse survival was monitored and plotted. Student t test and Log-rank test were used to generate p values comparing the dual and triple combinations. n = 10 mice per group per treatment arm. (c-d) JeKo-1 tumor volume in untreated mice (circles), after treatment with rituximab and venetoclax (Ven + Rit, squares) or with rituximab and venetoclax in combination with BI-1206 (Ven + Rit + BI-1206, triangles) at day 14 after treatment initiation. Venetoclax (100 mg/kg) was given daily for 14 days. Rituximab (10 mg/kg) and BI-1206 (10 mg/kg) were given twice a week for 14 days. Treatment was initiated at day 14 post inoculation. Tumor volumes were measured at day 14 post treatment and mouse survival was monitored and plotted. Student t test and Log-rank test were used to generate p values comparing the dual and triple combos. n = 10 mice per group per treatment arm. (e-f) JeKo-1 tumor volume in untreated animals (circles), after treatment with rituximab and CHOP (CHOP + Rit, squares) or with rituximab and CHOP in combination with BI-1206 (CHOP + Rit + BI-1206, triangles) at day 8 after treatment initiation. For CHOP treatment, cyclophosphamide (C; 20 mg/kg), hydroxy doxorubicin (H; 1.65 mg/kg), oncovin (O; 0.25 mg/kg) was given daily for 14 days and prednisone (P; 0.1 mg/kg) was given 5 days per week for 14 days. Rituximab (10 mg/kg) a [file 13045_2022_1257_MOESM3_ESM.pptx]
