# Supplementary material for: Targeting FcγRIIB by antagonistic antibody BI-1206 improves the efficacy of rituximab-based therapies in aggressive mantle cell lymphoma
Source: J Hematol Oncol. 2022 Apr 11;15:42. doi: 10.1186/s13045-022-01257-9 (PMC8996600; doi:10.1186/s13045-022-01257-9)
Supplement: Supplementary file 2 — Additional file 2. Supplementary Figure S2. Efficacy assessment of BI-1206 monotherapy in an ibrutinib-venetoclax dual resistant MCL PDX model in vivo. (a) Schematic strategy of the experiment to assess in vivo efficacy in the PDX model. PDX-A cells were inoculated intravenously into NSG mice. At two weeks post cell inoculation, the mice (n = 10 per group) were randomly grouped and treated with isotype control (10 mg/kg, twice a week), BI-1206 (10 mg/kg, twice a week) or Rituximab (Rit, 10 mg/kg, twice a week) plus Lenalidomide (LEN, 2 mg/kg, daily). Peripheral blood was collected every two weeks. At the end of the experiment, the mice were euthanized, and peripheral blood was collected. (b) The mouse body weight was monitored every two weeks. (c-d) Peripheral blood was collected every two weeks and at the end of experiment, and subject to flow analysis by dual staining with CD5 and CD20 antibodies. The percentage of CD5+CD20+ cells representing MCL tumor cells were plotted (c). Human B2M levels in mouse serum were detected via B2M ELISA assay. (e-f) Lung (e) and kidney (f) were weighed. (g) Counts of lymph nodes were plotted. [file 13045_2022_1257_MOESM2_ESM.pptx]

## Slide 1
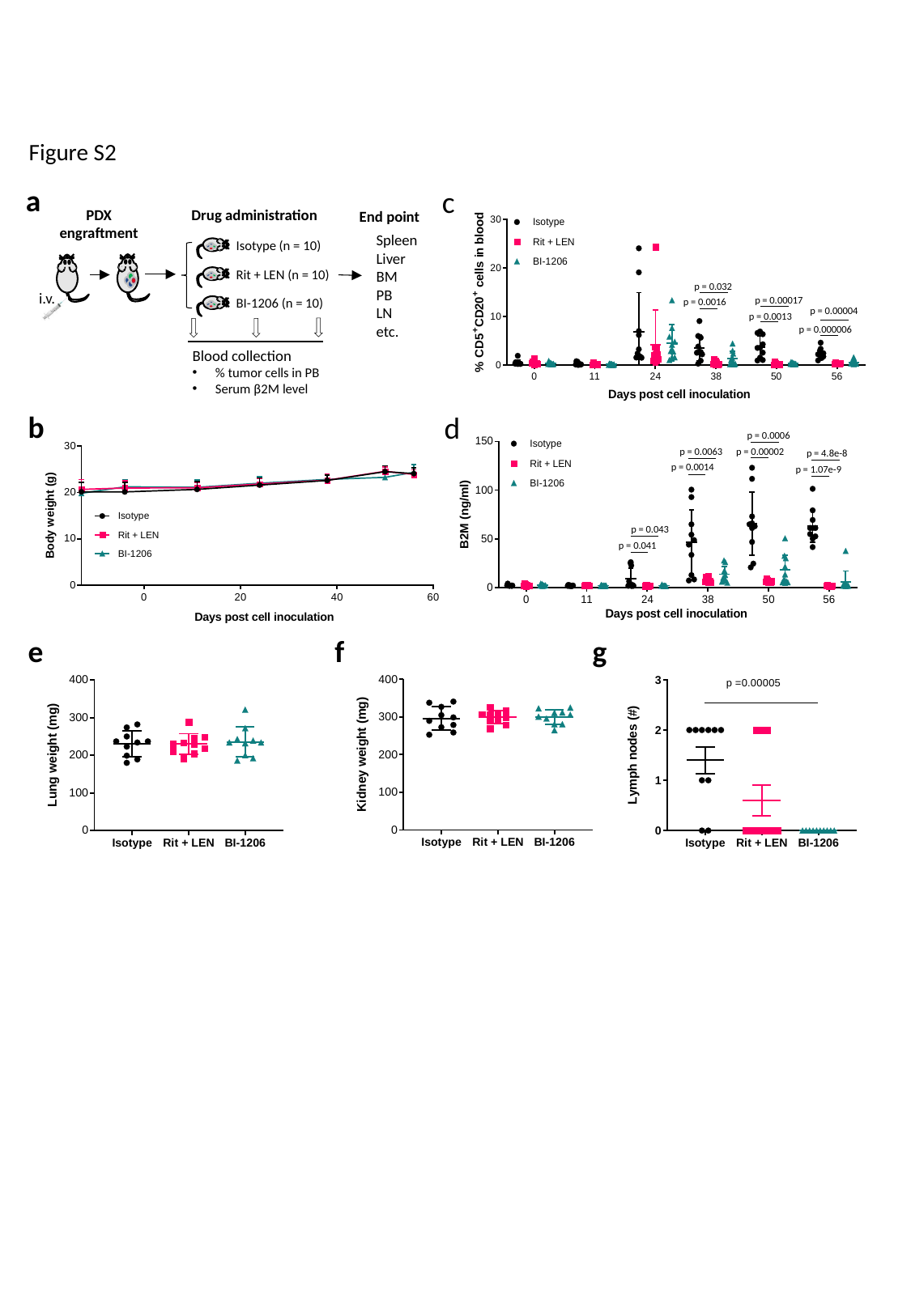

Figure S2
a
c
PDX engraftment
Drug administration
p = 0.032
p = 0.00017
p = 0.0016
p = 0.00004
p = 0.0013
p = 0.000006
End point
Spleen
Liver
BM
PB
LN
etc.
Isotype (n = 10)
Rit + LEN (n = 10)
i.v.
BI-1206 (n = 10)
Blood collection
% tumor cells in PB
Serum β2M level
b
d
p = 0.0006
p = 0.0063
p = 0.00002
p = 4.8e-8
p = 0.0014
p = 1.07e-9
p = 0.043
p = 0.041
e
f
g
